# Paradoxical Coronary Embolism as a Cause of Recurrent Myocardial Infarction: A Case Report

**DOI:** 10.5811/cpcem.52911

**Published:** 2026-04-21

**Authors:** Dago Berckmans

**Affiliations:** AZ Sint-Maarten, Department of Emergency Medicine, Mechelen, Belgium

**Keywords:** paradoxical embolism, coronary embolism, myocardial infarction, patent foramen ovale, case report

## Abstract

**Introduction:**

Paradoxical coronary embolism is a rare cause of myocardial infarction.

**Case Report:**

A 57-year-old man presented with acute chest pain after a recent non-ST elevation myocardial infarction, during which a patent foramen ovale was identified. On readmission, the electrocardiogram showed an inferior ST-elevation myocardial infarction, and angiography revealed a distal thrombotic occlusion in otherwise normal coronary arteries. No venous thromboembolism was found, but thrombophilia testing revealed heterozygous factor V Leiden. He was managed conservatively and underwent successful patent foramen ovale closure.

**Conclusion:**

This case highlights paradoxical embolism as a diagnostic consideration in acute myocardial infarction without coronary artery disease.

## INTRODUCTION

Most myocardial infarctions result from atherosclerotic plaque rupture, but in rare cases embolic occlusion is the culprit. When an embolus traverses a patent foramen ovale into the arterial circulation, the mechanism is termed paradoxical embolism. Coronary embolism is estimated to account for 3% of acute myocardial infarctions, most often due to infective endocarditis, atrial fibrillation, or valvular heart disease.^1^,^2^ Paradoxical embolism represents only a small fraction of these cases and is considered exceedingly rare.^1^,^3^ While patent foramen ovale is a well-established risk factor in cryptogenic stroke, its role in acute coronary syndromes is seldom reported.

We present a patient with recurrent myocardial infarction in the absence of coronary artery disease, in whom paradoxical embolism was strongly suspected. The case was further complicated by a transient neurological deficit, underscoring the systemic manifestations of paradoxical embolism and the importance of considering this diagnosis in the emergency department (ED).

## CASE REPORT

**Image 1:**
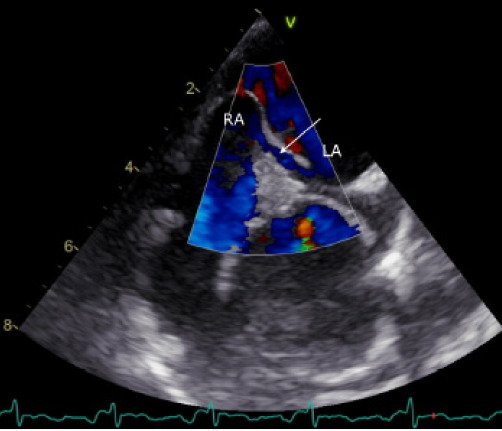
Transesophageal echocardiography, color Doppler mid-esophageal bicaval view. A long-tunnel patent foramen ovale (PFO) is seen with flow directed from the right atrium (RA) to the left atrium (LA). A white arrow marks the color Doppler jet across the PFO tunnel.

A 57-year-old man with a medical history of asthma and erosive gastritis had been admitted one week earlier with non-ST elevation myocardial infarction (NSTEMI). His cardiovascular risk profile included no smoking history, no hypertension, and no diabetes mellitus. He did have hypercholesterolemia and a strong family history, as his father had died of acute myocardial infarction at the age of 50. During the first admission, coronary angiography revealed no obstructive disease, but transesophageal echocardiography revealed a long-tunnel patent foramen ovale with color Doppler flow directed from the right atrium into the left atrium (Image 1, Video).

**Image 2:**
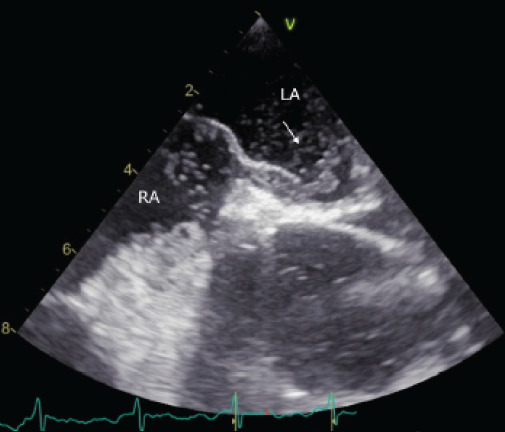
Transesophageal echocardiography, mid-esophageal bicaval view with agitated saline contrast. Microbubbles are visualized crossing from the right atrium (RA) to the left atrium (LA), confirming a large right-to-left shunt. A white arrow highlights the bubbles entering the left atrium.

An agitated saline contrast study confirmed a large right-to-left shunt, with microbubbles crossing from the right to left (Image 2). Left ventricular function was normal, and only minimal aortic root atheroma was seen.

The patient was discharged on aspirin and ticagrelor but presented again to the ED several days later with sudden retrosternal chest pain. He also described transient weakness of the left arm lasting approximately 30 minutes, which had resolved before arrival. He denied speech disturbance or visual symptoms. Upon arrival, his vitals were as follows: heart rate, 58 beats per minute; blood pressure, 185/99 millimeters of mercury; oxygen saturation, 98% on room air; and temperature normal. On examination, he was alert, oriented, and in no acute distress. Cardiopulmonary examination was unremarkable. Neurological evaluation revealed no residual deficits, with intact cranial nerves, full motor strength and sensation, normal coordination, symmetric reflexes, and no pronator drift.

**Image 3:**
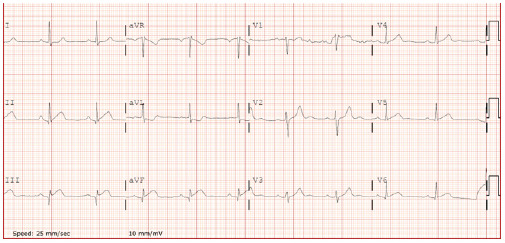
Electrocardiogram at presentation showing sinus rhythm with ST-segment elevation in leads II, III, and aVF, and reciprocal depression in aVL, consistent with acute inferior ST-elevation myocardial infarction.

Electrocardiogram showed sinus rhythm at 58 beats per minute with ST-segment elevation in leads II, III, and aVF, accompanied by reciprocal ST depression in aVL, consistent with inferior STEMI (Image 3).

Coronary angiography on this presentation revealed thrombotic occlusion of distal circumflex branches with an embolic morphology; the remainder of the coronary arteries were normal. No intervention was performed given the distal location. Left ventricular systolic function was overall preserved and appeared hyperdynamic, with a mid-inferior akinetic segment on left ventricular angiography, consistent with a recent inferior myocardial infarction. Noncontrast computed tomography of the brain was normal; magnetic resonance imaging (MRI) performed later revealed no acute ischemic lesions. Carotid duplex showed no significant stenosis. Lower extremity venous duplex revealed no deep vein thrombosis.

*CPC-EM Capsule*

What do we already know about this clinical entity?

*Paradoxical coronary embolism is a rare cause of myocardial infarction, typically linked to intracardiac shunts such as a patent foramen ovale*.

What makes this presentation of disease reportable?

*The absence of coronary atherosclerosis in recurrent infarction highlights an uncommon, non-atherosclerotic mechanism of myocardial injury*.

What is the major learning point?

*Normal coronary angiography does not exclude myocardial infarction and should prompt evaluation for nonatherosclerotic causes*.

How might this improve emergency medicine practice?

*Recognition of embolic mechanisms facilitates timely diagnostic evaluation, targeted management, and prevention of recurrent ischemic events*.

The patient was treated with dual antiplatelet therapy (aspirin, ticagrelor) and therapeutic low-molecular-weight heparin. His hospital course was uncomplicated. At discharge, he was transitioned to combination therapy with clopidogrel and rivaroxaban, with cardiology overseeing patent foramen ovale closure evaluation and hematology monitoring thrombophilia. Hematology later identified heterozygosity for factor V Leiden with an additional methylenetetrahydrofolate reductase variant. While the latter is of uncertain relevance in arterial events, the findings supported a prothrombotic predisposition.

## DISCUSSION

This case illustrates paradoxical coronary embolism as a rare but important cause of myocardial infarction. Several features support this diagnosis: angiographically normal proximal coronary arteries; abrupt distal occlusion with embolic morphology; and a known patent foramen ovale with a large right-to-left shunt. The patient’s cardiovascular risk profile included hypercholesterolemia and a strong family history of premature myocardial infarction, but he lacked other major risk factors such as smoking, hypertension, or diabetes. The first angiogram showed completely normal coronary arteries. On the second, the only abnormality was a thrombotic occlusion of distal circumflex branches with an appearance typical of embolization, while the proximal segments remained normal. This pattern made atherosclerotic plaque rupture unlikely.

The transient left-arm weakness raised suspicion of a concomitant cerebral embolic event. However, the brain MRI did not demonstrate any ischemic changes. Based on the clinical presentation and the absence of infarction on MRI, the episode most likely represented a transient ischemic attack.

The absence of deep vein thrombosis on duplex ultrasound raised the possibility of occult venous thrombosis (eg, pelvic veins), transient clot formation, or an underlying hypercoagulable state. A hematologic workup for thrombophilia demonstrated the presence of factor V Leiden and a methylenetetrahydrofolate reductase variant. Although the direct role of these abnormalities in arterial disease remains debated, they suggested a predisposition to thromboembolism.

The immediate priority is stabilization and treatment of the acute coronary syndrome. When the culprit thrombus is accessible, percutaneous coronary intervention (PCI)—including aspiration thrombectomy—may be considered, while systemic thrombolysis has occasionally been reported when PCI is not feasible.^4^ In distal lesions not amenable to intervention, as in our patient, conservative management with antiplatelet therapy and anticoagulation is reasonable, after which the focus should shift to identifying and addressing the underlying cause of the embolism. Secondary prevention may include a tailored duration of anticoagulation and, when a right-to-left shunt is implicated, consideration of patent foramen ovale closure. Although evidence is extrapolated from stroke trials, randomized data such as the Patent Foramen Ovale Closure or Anticoagulants versus Antiplatelet Therapy to Prevent Stroke Recurrence trial support closure in carefully selected patients.^5^ In the absence of formal guidelines, decisions should be individualized and coordinated through multidisciplinary consultation with cardiology, neurology, and hematology.

Comparable cases in the literature share a recurring pattern: a documented venous source or acute pulmonary embolism (PE), a right-to-left shunt across a patent foramen ovale, and angiographic evidence of coronary embolization. Hakim et al described paradoxical coronary embolism in the setting of upper-extremity deep vein thrombosis from a peripherally inserted central catheter with concomitant PE.^6^ Ferreira et al reported an inferior STEMI occurring in the setting of PE in a patient with a proven patent foramen ovale.^7^ Boberg et al similarly presented the triad of acute pulmonary embolism, a large patent foramen ovale with right-to-left shunt, and subsequent embolic occlusion of the right coronary artery.^8^ In contrast, our patient had neither deep vein thrombosis nor PE but was found to have an underlying thrombophilia, suggesting paradoxical coronary embolism may occur even in the absence of demonstrable venous thromboembolism.

## CONCLUSION

Paradoxical coronary embolism should be considered in patients presenting with STEMI and angiographically normal coronary arteries, particularly when a patent foramen ovale or transient neurologic symptoms are present. In the emergency department, priorities are stabilization and guideline-based acute coronary syndrome care with early multidisciplinary involvement to pursue the embolic source, tailor antithrombotic therapy, and consider patent foramen ovale closure for secondary prevention.

## Supplementary Information

Video Transesophageal echocardiography, color Doppler mid-esophageal view, with bicaval orientation demonstrating a long-tunnel patent foramen ovale with right-to-left shunting from the right to the left atrium.
